# Three subsets of sequence complexity and their relevance to biopolymeric information

**DOI:** 10.1186/1742-4682-2-29

**Published:** 2005-08-11

**Authors:** David L Abel, Jack T Trevors

**Affiliations:** 1 Director, The Gene Emergence Project, The Origin-of-Life Foundation, Inc., 113 Hedgewood Dr., Greenbelt, MD 20770-1610 USA; 2 Professor, Department of Environmental Biology, University of Guelph, Rm 3220 Bovey Building, Guelph, Ontario, N1G 2W1, Canada

**Keywords:** Self-organization, self-assembly, self-ordering, self-replication, genetic code origin, genetic information, self-catalysis.

## Abstract

Genetic algorithms instruct sophisticated biological organization. Three qualitative kinds of sequence complexity exist: random (RSC), ordered (OSC), and functional (FSC). FSC alone provides algorithmic instruction. Random and Ordered Sequence Complexities lie at opposite ends of the same bi-directional sequence complexity vector. Randomness in sequence space is defined by a lack of Kolmogorov algorithmic compressibility. A sequence is compressible because it contains redundant order and patterns. Law-like cause-and-effect determinism produces highly compressible order. Such forced ordering precludes both information retention and freedom of selection so critical to algorithmic programming and control. Functional Sequence Complexity requires this added programming dimension of uncoerced selection at successive decision nodes in the string. Shannon information theory measures the relative degrees of RSC and OSC. Shannon information theory cannot measure FSC. FSC is invariably associated with all forms of complex biofunction, including biochemical pathways, cycles, positive and negative feedback regulation, and homeostatic metabolism. The algorithmic programming of FSC, not merely its aperiodicity, accounts for biological organization. No empirical evidence exists of either RSC of OSC ever having produced a single instance of sophisticated biological organization. Organization invariably manifests FSC rather than successive random events (RSC) or low-informational self-ordering phenomena (OSC).

## Background

"Linear complexity" has received extensive study in many areas relating to Shannon's syntactic transmission theory [1-3]. This theory pertains only to engineering. Linear complexity was further investigated by Kolmogorov, Solomonoff, and Chaitin [4-8]. Compressibility became the measure of linear complexity in this school of thought. Hamming pursued Shannon's goal of noise-pollution reduction in the engineering communication channel through redundancy coding [9].

Little progress has been made, however, in measuring and explaining *intuitive information*. This is especially true regarding the derivation through natural process of semantic instruction. The purely syntactic approaches to sequence complexity of Shannon, Kolmogorov, and Hamming have little or no relevance to "meaning." Shannon acknowledged this in the 3^rd ^paragraph of his first famous paper right from the beginning of his research [2]. The inadequacy of more recent attempts to define and measure functional complexity [10-45] will be addressed in a separate manuscript.

Nucleic acid instructions reside in linear, digital, resortable, and unidirectionally read sequences [46-49]. Replication is sufficiently mutable for evolution, yet conserved, competent, and repairable for heritability [50]. An exception to the unidirectionality of reading is that DNA can occasionally be read from both directions simultaneously. For example, the circular bacterial chromosome can be replicated in both directions at the same time [51] But the basic principle of unidirectionality of the linear digital flow of information nonetheless remains intact.

In life-origin science, attention usually focuses on a theorized pre-RNA World [52-55]. RNA chemistry is extremely challenging in a prebiotic context. Ribonucleotides are difficult to activate (charge). And even oligoribonucleotides are extremely hard to form, especially without templating. The maximum length of such single strands in solution is usually only eight to ten monomers (mers). As a result, many investigators suspect that some chemical RNA analog must have existed [56,57]. For our purposes here of discussing linear sequence complexity, let us assume adequate availability of all four ribonucleotides in a pre-RNA prebiotic molecular evolutionary environment. Any one of the four ribonucleotides could be polymerized next in solution onto a forming single-stranded polyribonucleotide. Let us also ignore in our model for the moment that the maximum achievable length of aqueous polyribonucleotides seems to be no more than eight to ten monomers (mers). Physicochemical dynamics do not determine *the particular sequencing *of these single-stranded, untemplated polymers of RNA. The selection of the initial "sense" sequence is largely free of natural law influences and constraints. *Sequencing is dynamically inert *[58]. Even when activated analogs of ribonucleotide monomers are used in eutectic ice, incorporation of both purine and pyrimidine bases proceed at comparable rates and yields [59]. Monnard's paper provides additional evidence that *the sequencing *of untemplated single-stranded RNA polymerization in solution is dynamically inert – that the sequencing is not determined or ordered by physicochemical forces. Sequencing would be statistically unweighted given a highly theoretical "soup" environment characterized by 1) equal availability of all four bases, and 2) the absence of complementary base-pairing and templating (e.g., adsorption onto montmorillonite).

Initial sequencing of single-stranded RNA-like analogs is crucial to most life-origin models. Particular sequencing leads not only to a theorized self- or mutually-replicative primary structure, but to catalytic capability of that same or very closely-related sequence. One of the biggest problems for the pre-RNA World model is finding sequences that can *simultaneously *self-replicate and catalyze needed metabolic functions. For even the simplest protometabolic function to arise, large numbers of such self-replicative and metabolically contributive oligoribonucleotides would have to arise at the same place at the same time.

Little empirical evidence exists to contradict the contention that untemplated *sequencing *is dynamically inert (physically arbitrary). We are accustomed to thinking in terms of base-pairing complementarity determining sequencing. It is only in researching the pre-RNA world that the problem of single-stranded metabolically functional sequencing of ribonucleotides (or their analogs) becomes acute. And of course highly-ordered templated sequencing of RNA strands on natural surfaces such as clay offers no explanation for biofunctional sequencing. The question is never answered, "From what source did the *template *derive its functional information?" In fact, no empirical evidence has been presented of a naturally occurring inorganic template that contains anything more than combinatorial uncertainty. No bridge has been established between combinatorial uncertainty and utility of any kind.

It is difficult to polymerize even activated ribonucleotides without templating. Eight to ten mers is still the maximum oligoribonucleotide length achievable in solution. When we appeal to templating as a means of determining sequencing, such as adsorption onto montmorillonite, physicochemical determinism yields highly ordered sequencing (e.g., polyadenines) [60]. Such highly-ordered, low-uncertainty sequences retain almost no prescriptive information. Empirical and rational evidence is lacking of physics or chemistry determining semantic/semiotic/biomessenger functional sequencing.

Increased frequencies of certain ribonucleotides, CG for example, are seen in *post-textual *reference sequences. This is like citing an increased frequency of "qu" in post-textual English language. The only reason "q" and "u" have a higher frequency of association in English is because of arbitrarily chosen rules, not laws, of the English language. Apart from linguistic rules, all twenty-six English letters are equally available for selection at any sequential decision node. But we are attempting to model a purely pre-textual, combinatorial, chemical-dynamic *theoretical *primordial soup. No evidence exists that such a soup ever existed. But assuming that all four ribonucleotides might have been equally available in such a soup, no such "qu" type rule-based linkages would have occurred chemically between ribonucleotides. They are freely resortable apart from templating and complementary binding. Weighted means of each base polymerization would not have deviated far from p = 0.25.

When we introduce ribonucleotide availability realities into our soup model, we would not expect hardly any cytosine to be incorporated into the early genetic code. Cytosine is extremely difficult even for highly skilled chemists to generate [61,62]. If an extreme paucity of cytosine existed in a primordial environment, uncertainty would have been greatly reduced. Heavily weighted means of relative occurrence of the other three bases would have existed. The potential for recordation of prescriptive information would have been reduced by the resulting low uncertainty of base "selection." All aspects of life manifest extraordinarily high quantities of prescriptive information. Any self-ordering (law-like behavior) or weighted-mean tendencies (reduced availability of certain bases) would have limited information retention.

If non-templated *dynamic chemistry *predisposes higher frequencies of certain bases, how did so many highly-informational genes get coded? Any programming effort would have had to fight against a highly prejudicial self-ordering dynamic redundancy. There would have been little or no uncertainty (bits) at each locus. Information potential would have been severely constrained.

### Genetic sequence complexity is unique in nature

"Complexity," even "sequence complexity," is an inadequate term to describe the phenomenon ofgenetic "recipe." Innumerable phenomena in nature are self-ordered or complex without being instructive (e.g., crystals, complex lipids, certain polysaccharides). Other complex structures are the product of digital recipe (e.g., antibodies, signal recognition particles, transport proteins, hormones). Recipe specifies algorithmic function. Recipes are like programming instructions. They are strings of prescribed decision-node configurable switch-settings. If executed properly, they become like bug-free computer programs running in quality operating systems on fully operational hardware. The cell appears to be making its own choices. Ultimately, everything the cell does is programmed by its hardware, operating system, and software. Its responses to environmental stimuli seem free. But they are merely pre-programmed degrees of operational freedom.

The digital world has heightened our realization that virtually all information, including descriptions of four-dimensional reality, can be reduced to a linear digital sequence. Most attempts to understand intuitive information center around description and knowledge [41,63-67]. Human epistemology and agency invariably get incorporated into any model of semantics. Of primary interest to The Gene Emergence Project, however, is the derivation through natural process of what Abel has called *prescriptive information *(semantic instruction; linear digital recipe; cybernetic programming) [68-71]. The rise of prescriptive information presumably occurred early in the evolutionary history of life. Biopolymeric messenger molecules were *instructing biofunction *not only long before *Homo sapiens *existed, but also long before metazoans existed. Many eubacteria and archaea depend upon nearly 3,000 highly coordinated genes. Genes are linear, digital, cybernetic sequences. They are meaningful, pragmatic, physically instantiated recipes.

One of the requirements of any semantic/semiotic system is that the selection of alphanumeric characters/units be "arbitrary"[47]. This implies that they must be contingent and independent of causal determinism. Pattee [72-74] and Rocha [58] refer to this arbitrariness of sequencing as being "dynamically inert." "Arbitrary" does not mean in this context "random," but rather "unconstrained by necessity." Contingent means that events could occur in multiple ways. The result could just as easily have been otherwise. Unit selection at each locus in the string is unconstrained. The laws of physics and chemistry apply equally to whatever sequencing occurs. The situation is analogous to flipping a "fair coin." Even though the heads and tails side of the coin are physically different, the outcome of the coin toss is unrelated to dynamical causation. A heads result (rather than a tails) is contingent, unconstrained by initial conditions or law.

No law of physics has utility without insertion of a *symbolic representation *of the initial conditions. This usually comes in the form of measurement or graph coordinates. The initial physical conditions themselves cannot be inserted into a mathematical formula. Only a mathematical *representation *can be inserted. Physicist Howard Pattee refers to this as a "description" of initial conditions. The "epistemic cut" [75,76], "Complementarity" [77-81], and "Semantic Closure" [82-85] must occur between physicality and any description of dynamics such as the tentative formal generalizations we call laws.

Pattee's Epistemic Cut, Complementarily, and Semantic Closure apply equally well to sequences of physical symbol vehicles [72-75,77-80,84,86-89]. Nucleotides and their triplet-codon "block codes" *represent *each amino acid. Genes are informational messenger molecules specifically because codons function as semantic physical symbol vehicles. A codon "means" a certain amino acid. The instantiation of prescriptive information into biopolymers requires an arbitrary reassortment potential of these symbol vehicles in the linear sequence. This means that sequencing is dynamically inert. If the sequence were ordered by law-like constraint, the sequence would manifest monotonous redundancy of monomer occurrence. There would be little or no uncertainty at each decision node. Uncertainty (contingency: freedom from necessity) is required in a physical matrix for it to serve as a vehicle of descriptive or prescriptive information.

### Sequence complexity falls into three qualitative categories

1. Random Sequence Complexity (RSC),

2. Ordered Sequence Complexity (OSC), and

3. Functional Sequence Complexity (FSC)

**Figure 1:**
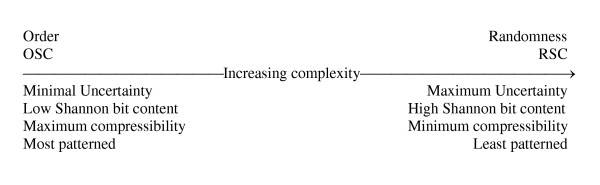
The inverse relationship between order and complexity as demonstrated on a linear vector progression from high order toward greater complexity (modified from [93]).

Sequence order and complexity are at opposite ends of a bi-directional vector (Fig. 1). *The most complex sequence is a random sequence with no recognizable patterns or order*. Shannon uncertainty is a function of -log_2 _p when decision nodes offer equiprobable and independent choice opportunities. Maximum sequence order has a probability of 1.0 at each locus in the string. A polyadenine, for example, has a probability of nearly 1.0 of having an adenine occur at any given four-way decision-node locus in the string. P = 1.0 represents 0 uncertainty. Minimum sequence order (maximum complexity; sequence randomness) has a probability of 0.5 at each binary node. In a binary system, P = 0.5 represents maximum uncertainty (1.0 bit at that binary decision node). The above points have been clearly established by Gregory Chaitin [6,90,91] and Hubert Yockey [46-49,92-96].

#### Random Sequence Complexity (RSC)

*A linear string of stochastically linked units, the sequencing of which is dynamically inert, statistically unweighted, and is unchosen by agents; a random sequence of independent and equiprobable unit occurrence*.

Random sequence complexity can be defined and measured solely in terms of probabilistic combinatorics. Maximum Shannon uncertainty exists when each possibility in a string (each alphabetical symbol) is equiprobable and independent of prior options. When possibilities are not equiprobable, or when possibilities are linked (e.g., paired by association, such as "qu" in the rules of English language), uncertainty decreases. The sequence becomes less complex and more ordered because of redundant patterning, or because of weighted means resulting from relative unit availability. Such would be the case if nucleotides were not equally available in a "primordial soup." This is demonstrated below under the section labeled "Ordered Sequence Complexity (OSC)."

Random sequence complexity (RSC) has four components:

1. The number of "symbols" in the "alphabet" that could potentially occupy each locus of the sequence (bit string)

(e.g., four potential nucleotide "alphabetical symbols" could occupy each monomeric position in a forming polynucleotide. In the English language, there are 26 potential symbols excluding case and punctuation.)

2. Equal probabilistic availability (often confused with post-selection frequency) of each "symbol" to each locus

(e.g., the availability of adenine was probably not the same as that of guanine, cytosine, or uracil to each position in a randomly forming primordial oligoribonucleotide. When each possibility is not equiprobable, weighted means must be used to calculate uncertainty. See equation 1)

3. The number of loci in the sequence

(e.g., the number of ribonucleotides must be adequate for a ribozyme to acquire minimal happenstantial function. A minimum of 30–60 "mers" has been suggested [97,98]

4. Independence of each option from prior options.

(e.g., in the English language, the letters "qu" appear together with much higher frequency than would be expected from independent letter selections where P = 1/26. Thus, if the generation of the signal were viewed as a stationary Markov process [as Shannon transmission theory does], conditional probabilities would have to be used to calculate the uncertainty of the letter "u".)

The Shannon uncertainty of random alphanumeric symbol sequences can be precisely quantified. No discussions of "aboutness" [12,13,99] or "before and after" differences of "knowledge" [100-104] are relevant to a measure of the Shannon *uncertainty *of RSC. Sequences can be quantitatively compared with respect to syntax alone.

In computer science, "bits" refer generically to "the number of binary switch-setting opportunities" in a computational algorithm. Options are treated as though they were equiprobable and independent combinatorial possibilities. Bits are completely nonspecific about which particular selection is made at any switch. The size of the program is measured in units of RSC. But the programming decisions at each decision node are anything but random.

Providing the information of how each switch is set is the very essence of what we want when we ask for instructions. The number of bits or bytes in a program fails to provide this intuitive meaning of information. The same is true when we are told that a certain gene contains X number of megabytes. Only the specific reference sequences can provide the prescriptive information of that gene's instruction. Measurements of RSC are not relevant to this task.

#### Ordered Sequence Complexity (OSC)

*A linear string of linked units, the sequencing of which is patterned either by the natural regularities described by physical laws (necessity) or by statistically weighted means (e.g., unequal availability of units), but which is not patterned by deliberate choice contingency (agency)*.

Ordered Sequence Complexity is exampled by a dotted line and by polymers such as polysaccharides. OSC in nature is so ruled by redundant cause-and-effect "necessity" that it affords the least complexity of the three types of sequences. The mantra-like matrix of OSC has little capacity to retain information. OSC would limit so severely information retention that the sequence could not direct the simplest of biochemical pathways, let alone integrated metabolism.

**Figure 2:**
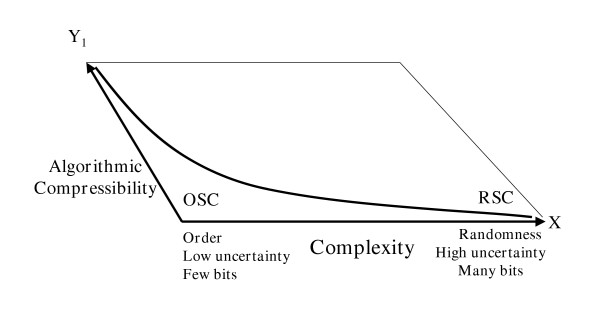
The adding of a second dimension to Figure 2 allows visualization of the relationship of Kolmogorov algorithmic compressibility to complexity. The more highly ordered (patterned) a sequence, the more highly compressible that sequence becomes. The less compressible a sequence, the more complex is that sequence. A random sequence manifests no Kolmogorov compressibility. This reality serves as the very definition of a random, highly complex string.

Appealing to "unknown laws" as life-origin explanations is nothing more than an appeal to cause-and-effect necessity. The latter only produces OSC with greater order, less complexity, and less potential for eventual information retention (Figs. 1 and 2).

The Shannon uncertainty equation would apply if forming oligoribonucleotides were stochastic ensembles forming out of sequence space:



where M = 4 ribonucleotides in an imagined "primordial soup." Suppose the prebiotic availability p_i _for adenine was 0.46, and the p_i _'s for uracil, guanine, and cytosine were 0.40, 0.12, and 0.02 respectively. This is being generous for cytosine, since cytosine would have been extremely difficult to make in a prebiotic environment [62]. Using these hypothetical base-availability probabilities, the Shannon uncertainty would have been equal to Table 1

**Table 1:** Hypothetical pre-biotic base availabilities

Adenine	0.46 (- log_2 _0.46)	=	0.515
Uracil	0.40 (- log_2 _0.40)	=	0.529
Guanine	0 12 (- log_2 _0.12)	=	0.367
Cytosine	0.02 (- log_2 _0.02)	=	0.113
	1.00		1.524 bits

Notice how unequal availability of nucleotides (*a form of ordering*) greatly reduces Shannon uncertainty (a measure of sequence complexity) at each locus of any biopolymeric stochastic ensemble (Fig. 1). Maximum uncertainty would occur if all four base availability probabilities were 0.25. Under these equally available base conditions, Shannon uncertainty would have equaled 2 bits per independent nucleotide addition to the strand. A stochastic ensemble formed under aqueous conditions of mostly adenine availability, however, would have had little information-retaining ability because of its high order.

Even less information-retaining ability would be found in an oligoribonucleotide adsorbed onto montmorillonite [60,97,105-108]. Clay surfaces would have been required to align ribonucleotides with 3' 5' linkages. The problem is that only polyadenines or polyuracils tend to form. Using clay adsorption to solve one biochemical problem creates an immense informational problem (e.g., high order, low complexity, low uncertainty, low information retaining ability, see Fig. 1). High order means considerable compressibility. The Kolmogorov [4] algorithmic compression program for clay-adsorbed biopolymers (Fig. 2) would read: "Choose adenine; repeat the same choice fifty times." Such a redundant, highly-ordered sequence could not begin to prescribe even the simplest protometabolism. Such "self-ordering" phenomena would not be the key to life's early algorithmic programming.

In addition to the favored RNA Word model [55,109], life origin models include clay life [110-113]; early three-dimensional genomes [114,115]; "Metabolism/Protein First" [116-119]; "Co-evolution" [120] and "Simultaneous nucleic acid and protein" [121-123]; and "Two-Step" models of life-origin [124-126]. In all of these models, "self-ordering" is often confused with "self-organizing." All known life depends upon genetic instructions. No hint of metabolism has ever been observed independent of an oversight and management information/instruction system. We use the term "bioengineering" for a good reason. Holistic, sophisticated, integrative processes such as metabolism don't just happen stochastically. Self-ordering in nature does. But the dissipative structures of Prigogine's chaos theory [127] are in a different category from the kind of "self-organization" that would be required to generate genetic instructions or stand-alone homeostatic metabolism. Semantic/semiotic/bioengineering function requires dynamically inert, resortable, physical symbol vehicles that represent time-independent, non-dynamic "meaning." (e.g., codons) [73,74,86,87,128-131].

No empirical or rational basis exists for granting to chemistry non-dynamic capabilities of functional sequencing. Naturalistic science has always sought to reduce chemistry to nothing more than dynamics. In such a context, chemistry cannot explain a *sequencing *phenomenon that is dynamically inert. If, on the other hand, chemistry possesses some metaphysical (beyond physical; beyond dynamics) transcendence over dynamics, then chemistry becomes philosophy/religion rather than naturalistic science. But if chemistry determined functional sequencing dynamically, sequences would have such high order and high redundancy that genes could not begin to carry the extraordinary prescriptive information that they carry.

Bioinformation has been selected algorithmically at the covalently-bound sequence level to instruct eventual three-dimensional shape. The shape is specific for a certain structural, catalytic, or regulatory function. All of these functions must be integrated into a symphony of metabolic functions. Apart from actually producing function, "information" has little or no value. No matter how many "bits" of possible combinations it has, there is no reason to call it "information" if it doesn't at least have the potential of producing something useful. What kind of information produces function? In computer science, we call it a "program." Another name for computer software is an "algorithm." No man-made program comes close to the technical brilliance of even Mycoplasmal genetic algorithms. Mycoplasmas are the simplest known organism with the smallest known genome, to date. How was its genome and other living organisms' genomes programmed?

#### Functional Sequence Complexity (FSC)

*A linear, digital, cybernetic string of symbols representing syntactic, semantic and pragmatic prescription; each successive sign in the string is a representation of a decision-node configurable switch-setting – a specific selection for function*.

FSC is a succession of algorithmic selections leading to function. Selection, specification, or signification of certain "choices" in FSC sequences results only from nonrandom selection. These selections at successive decision nodes cannot be forced by deterministic cause-and-effect necessity. If they were, nearly all decision-node selections would be the same. They would be highly ordered (OSC). And the selections cannot be random (RSC). No sophisticated program has ever been observed to be written by successive coin flips where heads is "1" and tails is "0."

We speak loosely as though "bits" of information in computer programs represented specific integrated binary choice commitments made with intent at successive algorithmic decision nodes. The latter is true of FSC, but technically such an algorithmic process cannot possibly be measured by bits (-log_2 _P) except in the sense of transmission engineering. Shannon [2,3] was interested in signal space, not in particular messages. Shannon *mathematics *deals only with averaged probabilistic combinatorics. *FSC requires a specification of the sequence of FSC choices*. They cannot be averaged without loss of prescriptive information (instructions).

Bits in a computer program measure only the number of binary choice *opportunities*. Bits do *not *measure or indicate *which specific choices *are made. Enumerating the specific choices that work is the very essence of gaining information (in the intuitive sense). When we buy a computer program, we are paying for sequences of integrated specific decision-node choice-commitments that we expect to work for us. The essence of the instruction is the enumeration of the sequence of *particular *choices. This necessity defines the very goal of genome projects.

Algorithms are *processes *or procedures that produce a needed result, whether it is computation or the end-products of biochemical pathways. Such strings of decision-node selections are anything but random. And they are not "self-ordered" by redundant cause-and-effect necessity. Every successive nucleotide is a quaternary "switch setting." Many nucleotide selections in the string are not critical. But those switch-settings that determine folding, especially, are highly "meaningful." Functional switch-setting sequences are produced only by uncoerced selection pressure. There is a cybernetic aspect of life processes that is directly analogous to that of computer programming. More attention should be focused on the reality and mechanisms of *selection at the decision-node level *of biological algorithms. This is the level of covalent bonding in primary structure. Environmental selection occurs at the level of post-computational halting. The fittest already-computed phenotype is selected.

We can hypothesize that metabolism "just happened," independent of directions, in a prebiotic environment billions of years ago. But we can hypothesize anything. The question is whether such hypotheses are plausible. Plausibility is often eliminated when probabilities exceed the "universal probability bound" [132]. The stochastic "self-organization" of even the simplest biochemical pathways is statistically prohibitive by hundreds of orders of magnitude. Without algorithmic programming to constrain (more properly "control") options, the number of possible paths in sequence space for each needed biopolymer is enormous. 10^15 ^molecules are often present in one test tube library of stochastic ensembles. But when multiple biopolymers must all converge at the same place at the same time to collectively interact in a controlled biochemically cooperative manner, faith in "self-organization" becomes "blind belief." No empirical data or rational scientific basis exists for such a metaphysical leap. Certainly no prediction of biological self-organization has been realized apart from SELEX-like bioengineering. SELEX is a selection/amplification methodology used in the engineering of new ribozymes [133-135]. Such investigator interference hardly qualifies as "self-organization." All of the impressive selection-amplification-derived ribozymes that have been engineered in the last fifteen years have been exercises in artificial selection, not natural selection.

Random sequences are the most complex (the least compressible). Yet empirical evidence of randomness producing sophisticated functionality is virtually nonexistent. Neither RSC nor OSC possesses the characteristics of informing or directing highly integrative metabolism. "Bits" of complexity alone cannot adequately measure or prescribe functional ("meaningful") bioinformation. Shannon information theory does not succeed in quantifying *the kind *of information on which life depends. It is called "information," but in reality we are quantifying only reduced combinatorial probabilistic uncertainty. This presupposes RSC. It is true that sophisticated bioinformation involves considerable complexity. But complexity is not synonymous with genetic instruction. Bioinformation exists as algorithmic programs, not just random combinations. And these programs require an operating system context along with common syntax and semantic "meanings" shared between source and destination.

The sequence of decision-node selections matters in how the polymer will finally fold. Folding is central to biofunction whether in a cell or a buffer in a test tube. In theory, the same protein can fold and unfold an infinite number of times via an ensemble of folding pathways [136]. But its favored minimal-free-energy molecular conformation is sequence dependent in the cell or assay mixture. The molecular memory for the conformation is the translated sequence. This is not to say that multiple sequences out of sequence space cannot assume the same conformation.

Nucleotides are grouped into triplet Hamming *block codes *[47], each of which *represents *a certain amino acid. No direct physicochemical causative link exists between codon and its *symbolized *amino acid in the physical translative machinery. Physics and chemistry do not explain why the "correct" amino acid lies at the opposite end of tRNA from the appropriate anticodon. Physics and chemistry do not explain how the appropriate aminoacyl tRNA synthetase joins a specific amino acid only to a tRNA with the correct anticodon on its opposite end.

**Figure 3:**
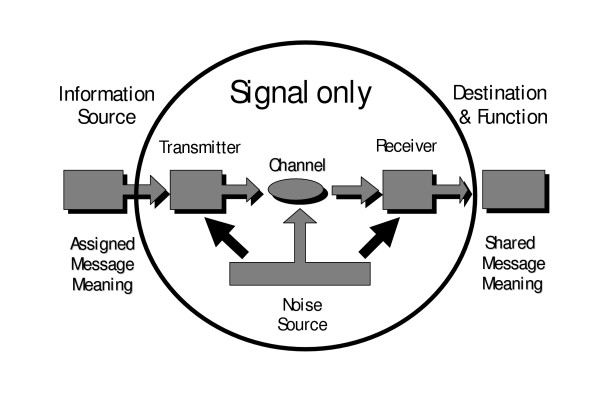
Shannon's original 1948 communication diagram is here modified with an oval superimposed over the limits of Shannon's actual research. Shannon never left the confines of this oval to address the essence of meaningful communication. Any theory of *Instruction *would need to extend outside of the oval to quantify the ideal function and indirect "meaning" of any message.

Genes are not analogous to messages; genes *are *messages. Genes are literal programs. They are sent from a source by a transmitter through a channel (Fig. 3) within the context of a viable cell. They are decoded by a receiver and arrive eventually at a final destination. At this destination, the instantiated messages catalyze needed biochemical reactions. Both cellular and extracellular enzyme functions are involved (e.g., extracellular microbial cellulases, proteases, and nucleases). Making the same messages over and over for millions to billions of years (relative constancy of the genome, yet capable of changes) is one of those functions. Ribozymes are also messages, though encryption/decryption coding issues are absent. The message has a destination that is part of a complex integrated loop of information and activities. The loop is mostly constant, but new Shannon information can also be brought into the loop via recombination events and mutations. Mistakes can be repaired, but without the ability to introduce novel combinations over time, evolution could not progress. The cell is viewed as an open system with a semi-permeable membrane. Change or evolution over time cannot occur in a closed system. However, DNA programming instructions may be stored in nature (e.g., in permafrost, bones, fossils, amber) for hundreds to millions of years and be recovered, amplified by the polymerase chain reaction and still act as functional code. The digital message can be preserved even if the cell is absent and non-viable. It all depends on the environmental conditions and the matrix in which the DNA code was embedded. This is truly amazing from an information storage perspective.

A noisy channel is one that produces a high corruption rate of the source's signal (Fig. 3). Signal integrity is greatly compromised during transport by randomizing influences. In molecular biology, various kinds of mutations introduce the equivalent of noise pollution of the original instructive message. Communication theory goes to extraordinary lengths to prevent noise pollution of signals of all kinds. Given this longstanding struggle against noise contamination of meaningful algorithmic messages, it seems curious that the central paradigm of biology today attributes genomic messages themselves solely to "noise."

Selection pressure works only on existing successful messages, and then only at the phenotypic level. Environmental selection does not choose which nucleotide to add next to a forming single-stranded RNA. Environmental selection is always after-the-fact. It could not have programmed primordial RNA genes. Neither could noise. Abel has termed this The GS Principle (Genetic Selection Principle) [137]. Differential molecular stability and happenstantial self- or mutual-replication are all that nature had to work with in a prebiotic environment. The environment had no goal or intent with which to "work." Wasted energy was just as good as "energy available for work" in a prebiotic world.

Denaturization factors like hydrolysis in water correspond to normal Second Law deterioration of the physical matrix of information retention. This results in the secondary loss of initial digital algorithmic integrity. This is another form of randomizing noise pollution of the prescriptive information that was instantiated into the physical matrix of nucleotide-selection sequences. But the particular physical matrix of retention should never be confused with abstract prescriptive information itself. The exact same message can be sent using completely different mass/energy instantiations. The Second Law operates on the physical matrix, not on the nonphysical conceptual message itself. The abstract message enjoys formal immunity from dynamic deterioration in the same sense that the mathematical laws of physics transcend the dynamics they model.

The purpose of biomessages is to produce and manage metabolic biofunctions, including the location, specificity, speed, and direction of the biochemical reactions. Any attempt to deny that metabolic pathways lack directionality and purpose is incorrect. Genes have undeniable "meaning" which is shared between source and destination (Fig. 3). Noise pollution of this "meaning" is greatly minimized by ideally optimized redundancy coding [9] and impressive biological repair mechanisms [138-143].

For prescriptive information to be conveyed, the destination must understand what the source meant in order to know what to do with the signal. It is only at that point that a Shannon signal becomes a bona fide message. Only shared meaning is "communication." This shared meaning occurs within the context of a relatively stable cellular environment, unless conditions occur that damage/injure or kill the cells. Considerable universality of "meaning" exists within biology since the Last Universal Common Ancestor (LUCA). For this reason, messages can be retrieved by bacteria even from the DNA of dead cells during genetic transformation [144]. The entire message is not saved, but significant "paragraphs" of recipe. The transforming DNA may escape restriction and participate in recombination events in the host bacterial cell. A small part of the entire genome message can be recovered and expressed. Evolution then proceeds without a final destination or direction.

Shannon's uncertainty quantification "H" is maximized when events are equiprobable and independent of each other. *Selection is neither*. Since choice with intent is fundamentally non probabilistic, each event is certainly not equiprobable. And the successive decision-node choice-commitments of any algorithm are never independent, but integrated with previous and future choices to collectively achieve functional success.

The "uncertainty" ("H") of Shannon is an epistemological term. It is an expression of our "surprisal" [145] or knowledge "uncertainty." But humans can also gain definite after-the-fact empirical knowledge of which specific sequences work. Such knowledge comes closer to "certainty" than "uncertainty." More often than not in everyday life, when we use the term "information," we are referring to a relative certainty of knowledge rather than uncertainty. Shannon equations represent a very limited knowledge system. But functional bioinformation is ontological, not epistemological. Genetic instructions perform their functions in objective reality independent of any knowers.

Stochastic ensembles could happenstantially acquire functional sequence significance. But a stochastic ensemble is more likely by many orders of magnitude to be useless than accidentally functional. Apart from nonrandom *selection *pressure, we are left with the statistical prohibitiveness of a purely chance metabolism and spontaneous generation. Shannon's uncertainty equations alone will never explain this phenomenon. They lack meaning, choice, and function. FSC, on the other hand, can be counted on to work. FSC becomes *the objective object *of our relative certainty. Its objective function becomes known empirically. Its specific algorithmic switch-settings are worth enumerating. We do this daily in the form of "reference sequences" in genome projects, applied pharmacology research, and genetic disease mapping. Specifically enumerated sequencing coupled with observed function is regarded as the equivalent of a proven "halting" program. This is the essence of FSC.

Symbols can be instantiated into physical symbol vehicles in order to manipulate dynamics to achieve physical utility. Symbol selections in the string are typically correlated into conceptually coordinated holistic utility by some externally applied operating system or language of arbitrary (dynamically inert) rules. But functional sequence complexity is always mediated through *selection *of each unit, not through chance or necessity.

The classic example of FSC is the nucleic acid algorithmic prescription of polyamino acid sequencing. Codon sequence determines protein primary structure only in a conceptual operational context. This context cannot be written off as a subjective epistemological mental construction of humans. Transcription, post-transcriptional editing, the translation operational context, and post-translational editing, all produced humans. The standard coding table has been found to be close to conceptually ideal given the relative occurrence of each amino acid in proteins [146]. A triplet codon is not a word, but an abstract conceptual block code for a protein letter [47]. Block coding is a creative form of redundancy coding used to reduce noise pollution in the channel between source and destination [9].

### Testable hypotheses about FSC

What testable empirical hypotheses can we make about FSC that might allow us to identify when FSC exists? In any of the following null hypotheses [137], demonstrating a single exception would allow falsification. We invite assistance in the falsification of any of the following null hypotheses:

#### Null hypothesis #1

*Stochastic *ensembles of physical units cannot program algorithmic/cybernetic function.

#### Null hypothesis #2

*Dynamically-ordered *sequences of individual physical units (physicality patterned by natural law causation) cannot program algorithmic/cybernetic function.

#### Null hypothesis #3

*Statistically weighted means *(e.g., increased availability of certain units in the polymerization environment) giving rise to patterned (compressible) sequences of units cannot program algorithmic/cybernetic function.

#### Null hypothesis #4

*Computationally successful *configurable switches cannot be set by chance, necessity, or any combination of the two, even over large periods of time.

We repeat that a single incident of nontrivial algorithmic programming success achieved without selection for fitness *at the decision-node programming level *would falsify any of these null hypotheses. This renders each of these hypotheses scientifically testable. We offer the prediction that none of these four hypotheses will be falsified.

The fundamental contention inherent in our three subsets of sequence complexity proposed in this paper is this: without volitional agency assigning meaning to each configurable-switch-position symbol, algorithmic function and language will not occur. The same would be true in assigning meaning to each combinatorial syntax segment (programming module or word). Source and destination on either end of the channel must agree to these assigned meanings in a shared operational context. Chance and necessity cannot establish such a cybernetic coding/decoding scheme [71].

How can one identify Functional Sequence Complexity empirically? *FSC can be identified empirically whenever an engineering function results from dynamically inert sequencing of physical symbol vehicles*. It could be argued that the engineering function of a folded protein is totally reducible to its physical molecular dynamics. But protein folding cannot be divorced from the causality of critical segments of primary structure sequencing. This sequencing was prescribed by the sequencing of Hamming block codes of nucleotides into triplet codons. This sequencing is largely dynamically inert. Any of the four nucleotides can be covalently bound next in the sequence. A linear digital cybernetic system exists wherein nucleotides function as representative symbols of "meaning." This particular codon "means" that particular amino acid, but not because of dynamical influence. No direct physicochemical forces between nucleotides and amino acids exist.

### The relationship between RSC, OSC, and FSC

**Figure 4:**
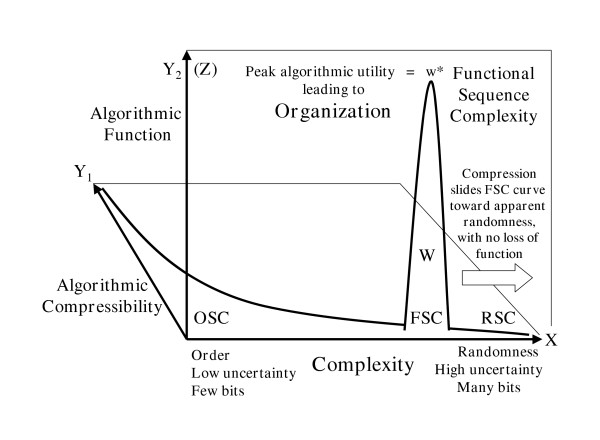
Superimposition of Functional Sequence Complexity onto Figure 2. The Y_1 _axis plane plots the decreasing degree of algorithmic compressibility as complexity increases from order towards randomness. The Y_2 _(Z) axis plane shows where along the same complexity gradient (X-axis) that highly instructional sequences are generally found. The Functional Sequence Complexity (FSC) curve includes all algorithmic sequences that work at all (W). The peak of this curve (w*) represents "what works *best*." The FSC curve is usually quite narrow and is located closer to the random end than to the ordered end of the complexity scale. Compression of an instructive sequence slides the FSC curve towards the right (away from order, towards maximum complexity, maximum Shannon uncertainty, and seeming randomness) *with no loss of function*.

A second dimension can be added to Figure 1, giving Figure 2, to visualize the relation of Kolmogorov algorithmic compression to order and complexity. Order and complexity cannot be combined to generate FSC. Order and complexity are at opposite ends of the same bi-directional vector. Neither has any direct relationship to cybernetic choices for utility. FSC *cannot *be visualized on the unidimensional vector of Figure 1. FSC cannot be visualized within Figure 2, either, despite its added dimension of Kolmogorov compressibility. At least a third dimension is required to visualize the functionality of each sequence. Figure 4 emphasizes the difference between algorithmic compression vs. *algorithmic function *produced by the sequence itself. Algorithmic compression is something we do *to *the sequence to shorten it. Algorithmic function is something *the sequence itself does *in an operational context.

Compression of language is possible because of repetitive use of letter and word combinations. Words correspond to reusable programming modules. The letter frequencies and syntax patterns of any language constrain a writer's available choices from among sequence space. But these constraints are the sole product of arbitrary intelligent choice within the context of that language. Source and destination reach a consensus of communicative methodology before any message is sent or received. This methodology is called a language or an operating system. Abstract concept ("choice contingency") determines the language system, not "chance contingency," and not necessity (the ordered patterning of physical "laws.")

Choice contingency is very different from chance contingency. Uncoerced (free) programming decisions have little in common with the self-ordering necessity of natural regularities. Neither necessity (OSC) nor chance (RSC) empirically displays any ability to instruct organization. At best, RSC and OSC per Prigogine's chaos theory [147] occasionally display self-ordering properties. "Self-ordering" is not the same as "self-organizing" despite abundant confusion in the literature. [148]. Organization requires Functional Sequence Complexity (FSC), not RSC or OSC. FSC in turn requires choice with algorithmic intent (in biology, selection for optimized biofunction and survivability).

What about some yet-to-be discovered "law" of nature? Couldn't that eventually explain FSC and the origin of life? Such a hope is based on a clouded understanding of a law and the dynamic "necessity" that a law describes. Laws are basically compression algorithms. Laws reduce reams of dynamic data down to parsimonious mathematical formulae. This is possible only because the behavior pattern is highly ordered, fixed, redundant, and low-informational. Degrees of freedom are severely constrained by necessity. But degrees of freedom are exactly what is required to generate FSC necessary for living organisms. Engineering decisions require freedom of selection. Environmental selection requires the same freedom. No undiscovered law can logically provide this needed dynamic decoherence and decoupling from law [149]. Decision nodes and configurable switches must be dynamically inert [58] with reference to their cybernetic function.

Controls are not the same as constraints. Controls cannot arise from self-ordering necessity where probability approaches 1.0 and uncertainty approaches 0 bits. Selection at decision nodes must be uncoerced to generate significant utility. Choice contingency realizes its maximum utility when options are equally available. Bits are maximized with 50:50 possibilities (unweighted means). Any natural-law constraints on selection only reduces bits, complexity, and algorithmic potential. Governance by any law would render flexible genetic control impossible. The organism's genome would lack freedom to respond to environmental changes. As a result, natural selection at the organism level would not be possible.

Proteins have a large number of decision nodes at which most of the twenty amino acids can be selected without total loss of protein function. Doesn't this fact negate the argument that life's recipe is digital and algorithmic? The answer is no. These sections of the protein Turing tape are merely non-interactive bends and buried sections of the highly-knotted globular protein. We would not expect all sections of the knotted protein to be critical to metabolic function. There are numerous other sections of the protein's sequence where only one or a few of the twenty amino acids can be used at each decision node. These sections contain the bulk of instructions. The other sections are somewhat like DNA introns that were once considered breaks between valued gene-product programming. The linear spacing within primary structure, after editing, is still critical. The exact messages have to occur at the right places in the one long protein molecule for it to fold and function properly. So instruction is inherent in the fact that the message segments must occur at positions 21–29, 67–71, 109–111, for example. *The spacing of catalytic segments is itself a critical part of the digital algorithmic program*.

### FSC quantitative units: The problem of measuring meaning

If there is any hope of quantifying meaning, we would need units with which to measure prescriptive information, not just weighted-mean combinatorial probabilities and mutual uncertainties. The "laws" of physics and chemistry are mathematical relationships made possible by fixed units of measure in equations scaled by constants. With Shannon uncertainty *as applied to computer science*, it is possible to have a fixed unit of measure only because each unit represents the constant value of one binary choice *opportunity*. Computer bits and bytes provide an additive function measuring the number of binary decision nodes. A bit does *not *represent "a choice" as is commonly believed. If that were true, it would not be a unit of constant measure. "Yes" does not equal "No." "On' does not have the same meaning value as "off." If it did, computation would be impossible. Choices at decision nodes matter only because they are different. So we know immediately that no one unit of measure could possibly be assigned to a decision-node switch-setting. At present, we can only quantify the number of decision nodes irrespective of which choice is made at each decision node.

We get away with the sloppy definition of "bit" in computer science (a binary "choice") only because we are measuring the "space" requirements needed for *any *program with that number of binary decisions. Bits measure averages, never specific choice commitments made with intent. The latter is the essence of algorithmic programming. Bit measurements are generic. They tell us nothing about which choice was made at each decision node. Bit measurements cannot tell us whether a program has a bug, or computes at all.

We live under the illusion that bits measure choices because we fail to recognize the role of background knowledge that we bring to the strict mathematical measurement of "bits." Our minds assign added information to the bit computation. This background information tells us that this particular bit value is for a certain program with a certain function. But the computation itself knows nothing of meaning or function. Knowledge about the particular message and its purpose is totally external to its bit value. Shannon knew this well. Not only did he carefully exclude all discussion of meaning and specific message function from his research [2], but later warned against regarding his probabilistic combinatorial "uncertainty" measures as "information theory" [150].

The function of an algorithmic program is often lost through attempts at reduction. Prescriptive information is lost in the process. Many computations are all-or-none ends in themselves. Just as random sequences cannot be compressed, computational algorithms cannot always be compressed.

FSC cannot have individual units of fixed value. Does this negate the reality of FSC? If so, we have a lot of explaining to do for fields such as engineering and computer science that depend squarely upon FSC. Language, rationality, and the scientific method itself all depend upon FSC, not RSC or OSC. Science must recognize that there are legitimate aspects of reality that cannot always be reduced or quantified.

## Conclusion

In summary, Sequence complexity can be 1) random (RSC), 2) ordered (OSC), or functional (FSC). OSC is on the opposite end of the bi-directional vectorial spectrum of complexity from RSC. FSC is usually paradoxically closer to the random end of the complexity scale than the ordered end. FSC is the product of nonrandom selection. FSC results from the equivalent of a succession of integrated algorithmic decision node "switch settings." FSC alone instructs sophisticated metabolic function. Self-ordering processes preclude both complexity and sophisticated functions. Self-ordering phenomena are observed daily in accord with chaos theory. But under no known circumstances can self-ordering phenomena like hurricanes, sand piles, crystallization, or fractals produce algorithmic organization. Algorithmic "self-organization" has never been observed [70] despite numerous publications that have misused the term [21,151-162]. Bone fide organization always arises from choice contingency, not chance contingency or necessity.

Reduced uncertainty (misnamed "mutual entropy") cannot measure prescriptive information (information that specifically informs or instructs). Any sequence that specifically informs us or prescribes how to achieve success inherently contains *choice *controls. The constraints of physical dynamics are not choice contingent. Prescriptive sequences are called "instructions" and "programs." They are not merely complex sequences. They are *algorithmically *complex sequences. They are cybernetic. Random sequences are maximally complex. But they don't *do *anything useful. Algorithmic instruction is invariably the key to any kind of sophisticated organization such as we observe in any cell. No method yet exists to quantify "prescriptive information" (cybernetic "instructions").

Nucleic acid prescription of function cannot be explained by "order out of chaos" or by "order on the edge of chaos" [163]. Physical phase changes cannot write algorithms. Biopolymeric matrices of high information retention are among the most complex entities known to science. They do not and can not arise from low-informational self-ordering phenomena. Instead of order from chaos, the genetic code was algorithmically optimized to deliver highly informational, aperiodic, specified complexity [164]. Specified complexity usually lies closer to the noncompressible unordered end of the complexity spectrum than to the highly ordered end (Fig. 4). Patterning usually results from the reuse of programming modules or words. But this is only secondary to choice contingency utilizing better efficiency. Order itself is not the key to prescriptive information.

The current usage of the word "complexity" in the literature represents a quagmire of confusion. It is an ill-defined, nebulous, often self-contradictory concept. We have defined FSC in a way that allows us to differentiate it from random and self-ordering phenomena, to frame testable empirical hypotheses about it, and to identify FSC when it exists.

Science has often progressed through the formulation of null hypotheses. Falsification allows elimination of plausible postulates [165,166]. The main contentions of this paper are offered in that context. We invite potential collaborators to join us in our active pursuit of falsification of these null hypotheses.

## Abbreviations used in this paper

Random Sequence Complexity (RSC); Ordered Sequence Complexity (OSC); Functional Sequence Complexity (FSC).
